# LDL Affects the Immunomodulatory Response of Endothelial Cells by Modulation of the Promyelocytic Leukemia Protein (PML) Expression via PKC

**DOI:** 10.3390/ijms24087306

**Published:** 2023-04-15

**Authors:** Kerrin Roos, Janine Berkholz

**Affiliations:** 1 Institute of Physiology, Charité-Universitätsmedizin, 10117 Berlin, Germany; 2 DZHK (German Centre for Cardiovascular Research), Partner Site Berlin, 10785 Berlin, Germany

**Keywords:** LDL, PKC, promyelocytic leukemia protein, IL-6, IL-8, endothelial cell

## Abstract

In addition to its function as an intravascular lipid transporter, LDL also triggers signal transduction in endothelial cells (ECs), which, among other things, trigger immunomodulatory cascades, e.g., IL-6 upregulation. However, the molecular mechanisms of how these LDL-triggered immunological responses in ECs are realized are not fully understood. Since promyelocytic leukemia protein (PML) plays a role in promoting inflammatory processes, we examined the relationship between LDL, PML, and IL-6 in human ECs (HUVECs and EA.hy926 cells). RT-qPCR, immunoblotting, and immunofluorescence analyses showed that LDL but not HDL induced higher PML expression and higher numbers of PML-nuclear bodies (PML-NBs). Transfection of the ECs with a *PML* gene-encoding vector or *PML*-specific siRNAs demonstrated PML-regulated IL-6 and IL-8 expression and secretion after LDL exposure. Moreover, incubation with the PKC inhibitor sc-3088 or the PKC activator PMA showed that LDL-induced PKC activity leads to the upregulation of *PML* mRNA and PML protein. In summary, our experimental data suggest that high LDL concentrations trigger PKC activity in ECs to upregulate PML expression, which then increases production and secretion of IL-6 and IL-8. This molecular cascade represents a novel cellular signaling pathway with immunomodulatory effects in ECs in response to LDL exposure.

## 1. Introduction

Lipoproteins are biochemically intensively characterized transporters of lipids (triacylglycerol, phospholipid, and cholesterol) in the aqueous blood compartment [1]. Five major classes of lipoproteins have been identified, which differ in their apolipoprotein and lipid content and composition (and hence their density and other physiochemical properties): chylomicrons, very low-density lipoprotein (VLDL), intermediate-density lipoprotein (IDL), low-density lipoprotein (LDL), and high-density lipoprotein (HDL).

Chylomicrons are fundamental for the distribution of ingested exogenous triacylglycerols within the body, while VLDLs transport the endogenous lipids formed after the partial oxidation of carbohydrates from the liver to the fat tissue where the triacylglycerols are taken up into the adipocytes. As a result of the breakdown of triacylglycerol, VLDLs convert to cholesterol-enriched IDLs and subsequently LDLs, which supply all cells in the periphery with cholesterol crucial for intact-cell-membrane assembly. HDLs, in turn, are responsible for scavenging excess cholesterol, which comes from precipitated LDLs or perished cells.

Properly controlled plasma concentrations of most lipoproteins are critical for their stable function in lipid transport [2]. About 30 mg/dL VLDL, 10 mg/dL IDL, and at least 50 mg/dL HDL are usually present in the plasma. For subjects with a low risk of developing atherosclerosis, LDL levels should be 100–115 mg/dL but not higher than 70 mg/dL for high-risk patients [3]. In contrast, the plasma levels of chylomicrons may vary significantly within a wide range depending on the amount of triacylglycerols ingested with food.

On the other hand, imbalanced lipoprotein levels might lead to the development of diseases, e.g., atherosclerosis as a consequence of LDL-dependent hypercholesterolemia, often in combination with hypertriglyceridemia [4,5]. An excessive plasma concentration of LDL cholesterol is widely recognized as a causal factor of endothelial dysfunction and atherosclerotic vascular diseases [6,7]. In particular, the LDL/HDL ratio appears to be relevant for the development of cardiovascular diseases: the higher this ratio, the higher the cardiovascular risk [8].

Besides their function as intravascular lipid transporters, it is becoming increasingly evident that lipoproteins induce immunomodulatory responses in endothelial cells (ECs) by binding to specific receptors [9]. Actually, several gene families of LDL receptors have been identified, each with unique biochemical properties and downstream signaling actions [10]. As a consequence, among others, more inflammatory cytokines such as interleukin 6 (IL-6) are expressed and secreted [11]. However, the molecular mechanisms of how these immunomodulatory responses are realized are still not fully understood.

Recently, it was found that promyelocytic leukemia protein (PML) plays a crucial role in inflammatory processes [12], e.g., by influencing IL-6 expression [13]. PML was originally identified to be a tumor-suppressor gene [14] but was later implicated in several other regulatory functions, e.g., in the cell cycle, apoptosis, or cellular stress responses [12]. PML is highly expressed, especially in ECs and inflamed tissues [15]. Recently, it has also been shown to be present in atherosclerotic plaques of human coronary arteries [16].

PML is also the main component of the so-called PML-nuclear bodies (PML-NBs) [17]. These multiprotein complexes have a diameter of 0.1–1 µm and occur in high numbers (10–30) in the nucleus of most cells [18]. In addition to the eponymous PML protein, around 150 other proteins have been identified, e.g., p53, Sp100, Daxx, and CBP, to participate in the self-organization of PML-NBs [19]. PML-NBs have different functions in cell physiology; they act as: (1) protein depots, (2) areas of nuclear activity, (3) sites of RNA regulation, or (4) hotspots for posttranslational modifications of proteins (e.g., SUMOylation) [12,20].

Because of its role in promoting inflammation, we hypothesized that PML is involved in LDL-mediated signaling in ECs, which line the inner surface of all blood vessels, and thus may be affected by circulating lipoproteins. To address this issue, we systematically studied the relationship between lipoproteins (especially LDL), PML, and other potentially involved factors in human ECs. As an outcome of our investigation, a previously unknown LDL-dependent signaling pathway in ECs was identified, involving the activation of protein kinase C (PKC) and upregulation of PML to increase the secretion of IL-6 and IL-8.

## 2. Results

### 2.1. LDL Induces IL-6 and IL-8 Secretion in Endothelial Cells

To evaluate whether lipoproteins induce the secretion of the cytokines IL-6 and IL-8 in ECs, ELISAs were performed on supernatants of EA.hy926 cells and HUVECs incubated either with lipoprotein mixtures (LPMix) supplemented with different concentrations of LDL (50 mg/dL = LPMix_LDL50, 100 mg/dL = LPMix_LDL100, 200 mg/dL = LPMix_LDL200) or with purified lipoprotein fractions at different concentrations (HDL 25 mg/dL, HDL 100 mg/dL, LDL 50 mg/dL, LDL 200 mg/dL) for either 3 h or 24 h.

First, the oxidized LDL (OxLDL) concentrations in the LPMix and the isolated lipoproteins fractions were quantified. Low OxLDL concentrations, which were found in all samples, are herein shown as relative OxLDL content related to the LDL concentrations (Supplemental Figure S1). Only non-significant differences were found between the samples.

**Figure 1:**
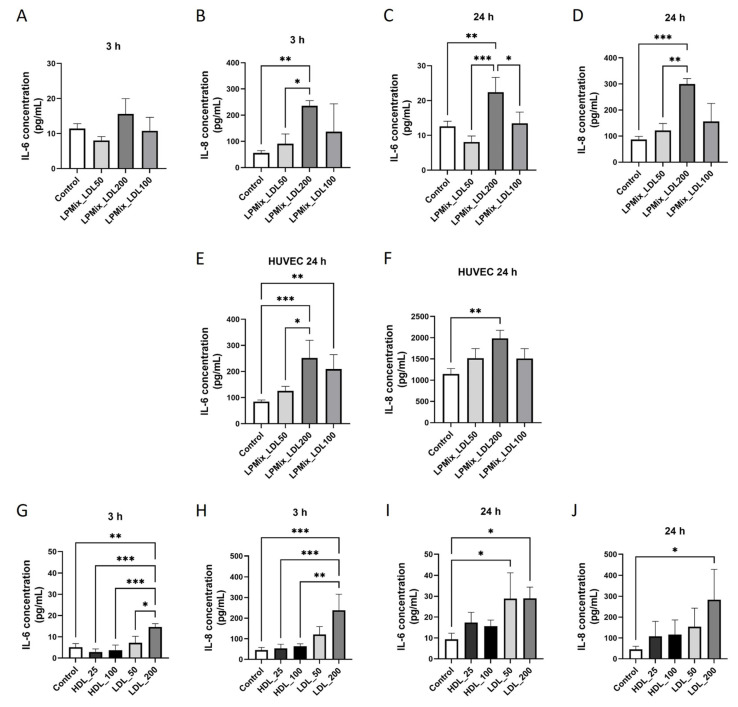
LDL increases the secretion of IL-6 and IL-8 in endothelial cells. (**A**–**D**) IL-6 (**A**,**C**) and IL-8 (**B**,**D**) protein levels in supernatants of EA.hy926 cells incubated with LPMix supplemented with LDL in different concentrations for 3 h (**A**,**B**) or 24 h (**C**,**D**), as quantified by ELISA. *n* = 3 (**A**,**C**), *n* = 4 (**B**,**D**), one-way ANOVA. (**E**,**F**) Quantification of IL-6 (**E**) and IL-8 (**F**) protein levels in supernatants of HUVECs incubated with LPMix supplemented with LDL in different concentrations for 24 h. *n* = 3, one-way ANOVA. (**G**–**J**) Determination of IL-6 (**G**,**I**) and IL-8 (**H**,**J**) protein levels in supernatants of EA.hy926 cells after incubation with high or low concentrations of isolated HDL or LDL for 3 h (**G**,**H**) or 24 h (**I**,**J**) by ELISA. *n* = 3 (**G**,**I**), *n* = 4 (**F**,**H**), one-way ANOVA. All graphs shown as mean ± SD, * *p* < 0.05, ** *p* < 0.01, *** *p* < 0.001.

As shown in Figure 1A,B, IL-6 secretion was only non-significantly different, whereas IL-8 secretion was higher (321%, *p* < 0.01) in EA.hy926 cells incubated with the LPMix_LDL200 for 3 h compared to control cells. In contrast, after 24 h incubation with LPMix_LDL200, IL-6 and IL-8 protein levels in the supernatant of EA.hy926 cells were increased by 78% and 244%, respectively (*p* < 0.01, *p* < 0.001; Figure 1C,D). In the supernatant of human umbilical vein endothelial cells (HUVECs), IL-6 and IL-8 levels were also higher (*p* < 0.01) after incubation with either the LPMix_LDL100 or the LPMix_LDL200 for 24 h (Figure 1E,F).

The experiments were also performed with purified HDL and LDL at different concentrations (Figure 1G–J). Incubation with 200 mg/dL LDL for 3 h resulted in 186% higher IL-6 (*p* < 0.01; Figure 1G) and 367% higher IL-8 (*p* < 0.001; Figure 1H) levels, respectively, compared to controls. EA.hy926 cells showed higher IL-6 secretion after 24 h incubation with 50 mg/dL LDL (208%, *p* < 0.05; Figure 1I) or 200 mg/dL LDL (209%, *p* < 0.05; Figure 1J). In contrast, IL-8 secretion was higher only in cells incubated with 200 mg/dL LDL for 24 h (515%, *p* < 0.05; Figure 1G). HDL administration had only a non-significant effect (*p* > 0.05) on the secretion of IL-6 or IL-8 at either 3 h or 24 h (Figure 1G–J). The data suggest that exposure of ECs with LDL but not HDL is accompanied with higher production and secretion rates of IL-6 and IL-8.

### 2.2. Stimulation of Endothelial Cells with High Doses of LDL Leads to Higher PML Expression

Because PML is also associated with inflammatory responses in ECs, the effect of the LPMix supplemented with different LDL concentrations on PML expression in HUVECs and EA.hy926 cells was investigated.

**Figure 2:**
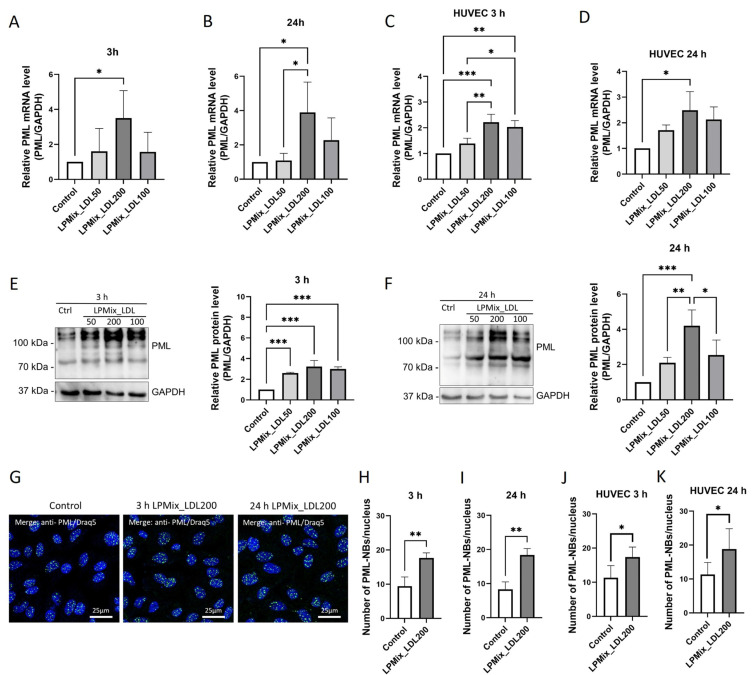
LDL added to lipoprotein mixtures dose-dependently regulates PML expression and PML-NB assembly in endothelial cells. (**A**–**D**) *PML* mRNA levels in EA.hy926 cells (**A**,**B**) or HUVECs (**C**,**D**) were quantified using RT-qPCR after incubation with LPMix supplemented with LDL in different concentrations for 3 h (**A**,**C**) or 24 h (**B**,**D**). Expression values relative to controls. *n* = 4, one-way ANOVA. (**E**,**F**) Immunoblotting on total lysates of EA.hy926 cells incubated with LPMix supplemented with LDL in different concentrations for 3 h (**E**) or 24 h (**F**). Expression values relative to controls. Representative immunoblots of *n* = 4, one-way ANOVA. (**G**) Immunocytochemistry with an anti-PML (green) antibody on EA.hy926 cells incubated with high concentrations of LDL (200 mg/dL) for 3 h or 24 h. Draq5 staining for labeling of cell nuclei (blue). Representative images of *n* = 3 experiments. (**H**–**K**) Number of PML-NBs per nucleus in EA.hy926 cells (**H**,**I**) or HUVECs (**J**,**K**) incubated with LPMix supplemented with high concentrations of LDL for 3 h (**H**,**J**) or 24 h (**I**,**K**). *n* = 3 Student’s *t*-tests. All graphs shown as mean ± SD, * *p* < 0.05, ** *p* < 0.01, *** *p* < 0.001.

As shown in Figure 2A,B, *PML* mRNA expression was higher in EA.hy926 cells incubated with LPMix_LDL200 for 3 h (250%, *p* < 0.05; Figure 2A) or 24 h (290%, *p* < 0.05; Figure 2B) than in the control cells. Incubation with LPMix_LDL100 or LPMix_LDL50 had only non-significant effects (*p* > 0.05) on *PML* mRNA expression at either 3 h or 24 h (Figure 2A,B). In HUVECs, *PML* mRNA levels were higher after incubation with LPMix_LDL100 or LPMix_LDL200 (Figure 2C,D).

To assess the effect of LPMix on PML protein expression in EA.hy926 cells, immunoblotting assays were performed on total cell lysates after 3 h or 24 h incubation (Figure 2E,F). These assays showed that 3 h incubation with any LPMix resulted in higher PML protein levels (161–232%, *p* < 0.001; Figure 2E) compared to control cells. Incubation of EA.hy926 cells with LPMix for 24 h resulted in higher PML protein levels only in cells incubated with LPMix_LDL200 (320%, *p* < 0.001; Figure 2F).

To quantify the number of PML-NBs in ECs incubated for 24 h with LPMix_LDL200, an immunofluorescence analysis was performed on EA.hy926 cells and HUVECs (Figure 2G). Consistent with the PML immunoblotting results, the average number of PML-NBs per nucleus was significantly higher (*p* < 0.05) in the cells incubated with the LPMix_LDL200 for 3 h or 24 h, both in EA.hy926 and HUVECs (EA.hy926: 67–88%, *p* < 0.01; HUVECs: 43–53%, *p* < 0.05; Figure 2H–K).

In summary, the incubation of ECs with LPMix, particularly supplemented with high concentrations of LDL, resulted in higher *PML* mRNA and PML protein levels and higher numbers of PML-NBs than in the control cells.

### 2.3. LDL Increases PML Levels in Endothelial Cells in a Dose-Dependent Manner

To characterize the effect of lipoproteins on PML expression in ECs in more detail, EA.hy926 cells were incubated with the purified lipoproteins LDL (LDL 50 mg/dL, LDL 200 mg/dL) or HDL (HDL 25 mg/dL, HDL 100 mg/dL) in different concentrations for either 3 h or 24 h.

**Figure 3:**
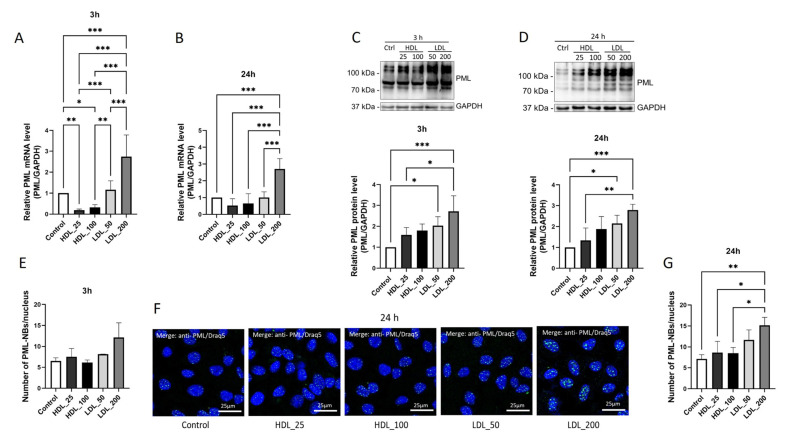
High doses of LDL increase PML expression and PML-NB assembly in endothelial cells. (**A**,**B**) *PML* mRNA levels in EA.hy926 cells were quantified by RT-qPCR after incubation with high or low concentrations of isolated HDL or LDL for 3 h (**A**) or 24 h (**B**). Expression values relative to control. n = 3 (**A**), *n* = 4 (**B**), one-way ANOVA. (**C**,**D**) Immunoblotting was performed on total lysates of EA.hy926 cells incubated with HDL or LDL in high or low concentrations for 3 h (**C**) or 24 h (**D**). Expression values relative to control. Representative immunoblots of *n* = 4, one-way ANOVA. (**E**) Representative images of EA.hy926 cells incubated with isolated HDL or LDL in high or low concentrations for 24 h and subjected to immunocytochemistry with an anti-PML (green) antibody and Draq5 staining for labeling of cell nuclei (blue). Representative images of *n* = 3 experiments. (**F**,**G**) Number of PML-NBs per nucleus in EA.hy926 cells incubated with isolated HDL or LDL in high or low concentrations for 3 h (**F**) or 24 h (**G**). *n* = 3, one-way ANOVA. All graphs shown as mean ± SD, * *p* < 0.05, ** *p* < 0.01, *** *p* < 0.001.

*PML* mRNA expression was lower when EA.hy926 cells were incubated with 25 mg/dL (−79%, *p* < 0.01) or 100 mg/dL (−68%, *p* < 0.05) of HDL for 3 h (Figure 3A) compared to controls. In contrast, incubation with 200 mg/dL of LDL for 3 h led to higher *PML* mRNA expression (175%, *p* < 0.05; Figure 3A) compared to controls. As shown in Figure 3B, 24 h incubation with HDL of both concentrations only led to a slight, non-significant (*p* > 0.05) decrease in *PML* mRNA levels, whereas incubation with 200 mg/dL of LDL caused *PML* mRNA levels to be higher by 171% (*p* < 0.001). Interestingly, 3 h of incubation led to higher PML protein levels when incubated with 50 mg/dL of LDL (+104%, *p* < 0.05; Figure 3C) or 200 mg/dL of LDL (+173%, *p* < 0.001; Figure 3C), whereas incubation with HDL showed no effect. Accordingly, incubation of EA.hy926 cells with isolated lipoproteins for 24 h led to higher PML protein levels when incubated with 50 mg/dL of LDL (+115%, *p* < 0.01; Figure 3D) or with 200 mg/dL of LDL (+180%; *p* < 0.0001; Figure 3D). Immunofluorescence analysis (Figure 3E) revealed that the average number of PML-NBs per nucleus was higher only in EA.hy.926 cells incubated with 200 mg/dL LDL for 24 h than in controls (+112%, *p* < 0.01; Figure 3F,G).

Taken together, these experimental data also suggest that LDL positively regulates PML expression and the formation of PML-NBs in ECs in a dose-dependent manner.

### 2.4. PML Mediates the LDL-Induced IL-6 and IL-8 Expression in ECs

**Figure 4:**
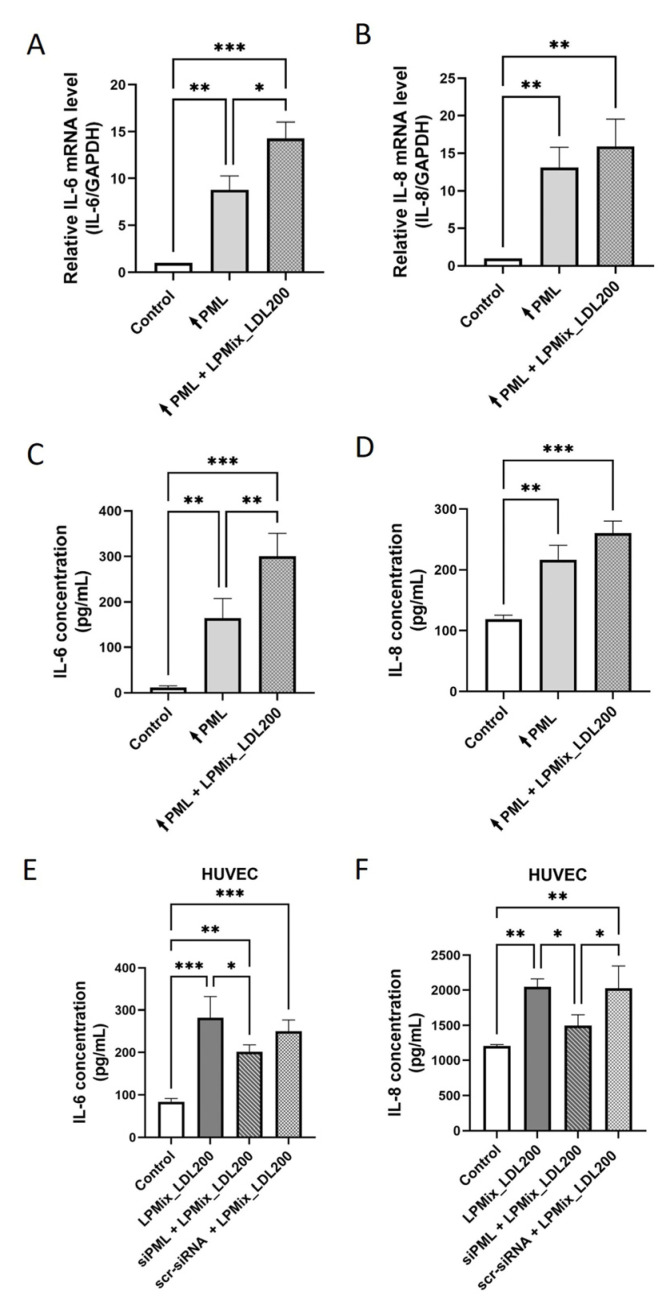
PML mediates LDL-triggered upregulation of IL-6 and IL-8 expression in endothelial cells. (**A**–**D**) *IL-6* (**A**) or *IL-8* (**B**) mRNA and IL-6 (**C**) or IL-8 (**D**) protein (ELISA on cell supernatants) levels were determined in EA.hy926 cells either transfected with a vector lacking a specific gene insert (control) or a pEGFP-C1-PML-IV-vector (↑PML) co-incubated without or with an LPMix supplemented with high LDL concentration for 24 h. *n* = 3, two-way ANOVA. (**E**,**F**) IL-6 (**E**) or IL-8 (**F**) protein levels in supernatants of HUVECs incubated with an LPMix supplemented with high LDL concentrations for 24 h with or without simultaneous transfection with either *PML*-specific siRNAs (siPML) or scrambled (scr) siRNAs (scr-siRNA)(control). *n* = 3, two-way ANOVA. All graphs shown as mean ± SD, * *p* < 0.05, ** *p* < 0.01, *** *p* < 0.001.

To find out whether the influence of LDL on IL-6 and IL-8 expression is mediated via PML, EA.hy926 cells were transfected with a pEGFP-C1-PML-IV vector or the control vector and incubated for 24 h with or without LPMix_LDL200 to be subsequently subjected to RT-qPCR analysis. As shown in Figure 4A, *PML*-transfected cells expressed more *IL-6* mRNA without exposure to LPMix_LDL200 (+783%, *p* < 0.01) or with exposure to LPMix_LDL200 (+1329%, *p* < 0.001) than the control-transfected cells. PML-overexpressing cells showed +1209% (*p* < 0.01; Figure 4B) higher *IL-8* mRNA expression than control cells. Additional incubation with LPMix_LDL200 was associated with +1593% (*p* < 0.01; Figure 4B) higher *IL-8* expression compared to control cells. Corresponding results were obtained if the IL-6 or IL-8 concentrations were quantified in the supernatants of EA.hy926 cells (Figure 4C,D).

Additional experimental evidence was obtained when HUVECs were incubated with LPMix_LDL200 and simultaneously transfected with *PML*-specific siRNAs or scrambled siRNAs. Here, the transfection of siPML resulted in either lower IL-6 or IL-8 secretion (−29% or −27%, *p* < 0.05, Figure 4E,F) compared to samples treated with the LPMix_LDL200 alone.

These findings suggest that the LDL-induced increase in IL-6 and IL-8 expression in ECs is PML influenced.

### 2.5. PKC Activity Is Higher in Endothelial Cells Exposed to Lipoproteins with High LDL Concentrations

**Figure 5:**
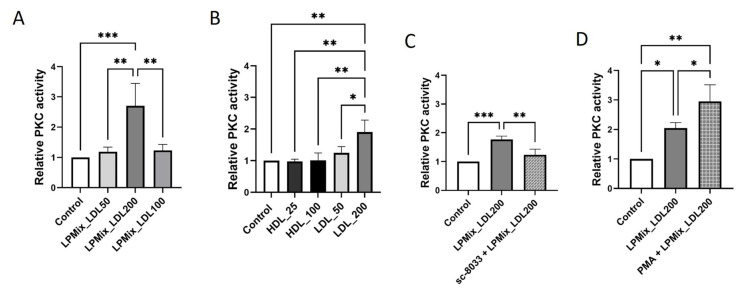
LDL increases PKC activity in endothelial cells. (**A**,**B**) PKC activity was determined using PKC activity assay kit on total lysates of EA.hy926 cells incubated with LPMix supplemented with LDL in different concentrations (**A**) or with high or low concentrations of purified HDL or LDL (**B**) for 24 h. Expression values relative to control. *n* = 3, one-way ANOVA. (**C**,**D**) Relative PKC activity in total lysates of EA.hy926 cells incubated with the LPMix supplemented with high LDL concentrations in the presence/absence of sc-3088 (1 µM) (**C**) or PMA (80 nM) (**D**) for 24 h, as quantified by ELISA. Expression values relative to control. *n* = 3, two-way ANOVA. All graphs shown as mean ± SD, * *p* < 0.05, ** *p* < 0.01, *** *p* < 0.001.

To find out whether PKC is involved in the signaling cascade triggered by LDL, a PKC activity assay was performed on total lysates of EA.hy926 cells incubated with the different LPMix (Figure 5A) or purified lipoproteins (Figure 5B). Incubation with LPMix_LDL200 led to 171% higher PKC activity (*p* < 0.001; Figure 5A) compared to controls. Accordingly, incubation with 200 mg/dL LDL alone resulted in 90% higher PKC activity (*p* < 0.01; Figure 5B) compared to control cells.

The PKC activity assay was also carried out on total lysates from EA.hy926 cells incubated with LPMix_LDL200, which were additionally treated with the PKC peptide inhibitor sc-3088 or the PKC activator phorbol 12-myristate 13-acetate (PMA) (Figure 5C,D). To confirm the biological activity of sc-3088 or PMA for subsequent experiments in ECs, EA.hy926 cells were first incubated with these modulators for 24 h to subsequently monitor the PKC activity (Supplementary Figure S2). As expected, incubation with sc-3088 resulted in a lower PKC activity (−37%, *p* < 0.01; Supplementary Figure S2A), whereas incubation with the PMA led to 36% higher PKC activity (*p* < 0.001; Supplementary Figure S2B) compared to the PKC activities in control cells.

As shown in Figure 5C, incubation with the combination of LPMix_LDL200 and sc-3088 for 24 h resulted in similar PKC activity as in control cells and in lower PKC activity (−30%, *p* < 0.01) compared to cells incubated with the LPMix_LDL200 alone. In contrast, simultaneous treatment of EA.hy926 cells with the LPMix_LDL200 and PMA resulted in higher PKC activity (+44%, *p* < 0.05; Figure 5D) compared to incubation with the LPMix_LDL200 alone.

These results demonstrated a regulatory association between lipoproteins and PKC activity in ECs.

### 2.6. LDL-Induced Upregulation of PML Is Mediated by PKC in Endothelial Cells

**Figure 6:**
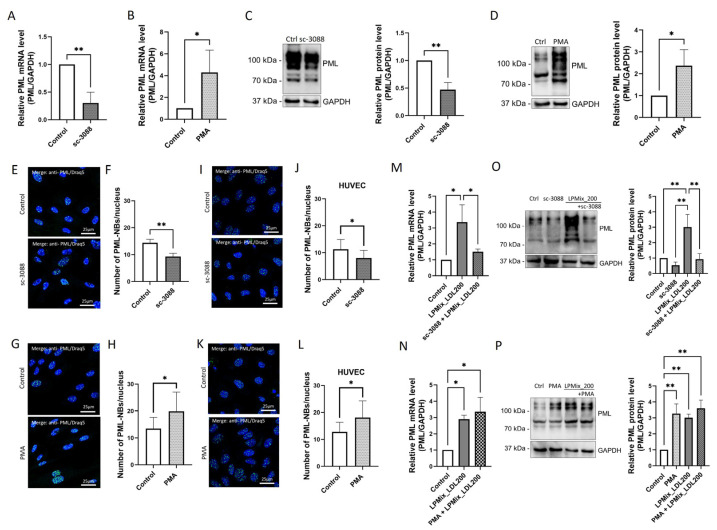
PKC regulates PML expression and PML-NB assembly in endothelial cells. (**A**,**B**) PML mRNA levels were determined using RT-qPCR on EA.hy926 cells incubated with the PKC inhibitor sc-3088 (**A**) or the PKC activator PMA (**B**) for 24 h. Expression values relative to control. *n* = 3, Student’s *t*-test. (**C**,**D**) Immunoblotting was performed to determine PML protein levels in total lysates of EA.hy926 cells that were either untreated (control) or incubated with sc-3088 (**C**) or PMA (**D**) for 24 h. Expression values relative to control. Representative immunoblots of *n* = 3, Student’s *t*-test. (**E**,**G**,**I**,**K**) Immunocytochemistry with an anti-PML (green) antibody on EA.hy926 cells (**E**,**G**) or HUVECs (**I**,**K**) incubated with sc-3088 (**E**,**I**) or PMA (**G**,**K**). Draq5 staining was performed for the labeling of cell nuclei (blue). Representative images of *n* = 3 experiments. (**F**,**H**,**J**,**L**) Number of PML-NBs per nucleus in EA.hy926 cells (**F**,**H**) or HUVECs (**J**,**L**) incubated with either sc-3088 (**F**,**J**) or PMA (**H**,**L**) for 24 h. *n* = 3, Student’s *t*-test. (**M**,**N**) RT-qPCR for the quantification of PML mRNA levels in EA.hy926 cells incubated with the LPMix with high LDL concentrations in the presence/absence of either sc-3088 (**M**) or PMA (**N**) for 24 h. Expression values relative to control. Representative immunoblots of *n* = 3, two-way ANOVA. (**O**,**P**) Immunoblotting for the determination of PML protein levels in total cell lysates of EA.hy926 cells incubated with the LPMix with high LDL concentrations in the presence or absence of either sc-3088 (**O**) or PMA (**P**) for 24 h. Expression values relative to control. *n* = 3, two-way ANOVA. All graphs shown as mean ± SD, * *p* < 0.05, ** *p* < 0.01.

Next, we investigated whether the PKC activity has an impact on PML expression. Therefore, EA.hy926 cells were first incubated with sc-3088 or PMA for 24 h to then be subjected to RT-qPCR (Figure 6A,B) or quantitative immunoblotting (Figure 6C,D). Incubation with sc-3088 led to lower *PML* mRNA levels (−70%, *p* < 0.01; Figure 6A), whereas incubation with PMA resulted in higher *PML* mRNA levels (+329%, *p* < 0.05; Figure 6B) compared to the control cells. As shown in Figure 6C,D, the PML protein levels were lower after incubation with sc-3088 for 24 h (−53%, *p* < 0.01; Figure 6C) while they were higher after incubation with PMA (136%, *p* < 0.05; Figure 6D) compared to the levels in the control cells. Moreover, as shown by immunofluorescence analysis (Figure 6E), incubation with sc-3088 resulted in a lower number of PML-NBs per nucleus (−36%, *p* < 0.01; Figure 6F). In contrast, incubation with PMA led to a higher number of PML-NBs per nucleus (+47%, *p* < 0.05; Figure 6G) compared to control cells. Similar results for the number of PML-NBs were obtained in HUVECs after treatment with sc-3088 or PMA (Figure 6H,I).

To determine whether the LDL-triggered induction of PML expression depends on PKC activity, *PML* mRNA and PML protein levels were quantified in EA.hy926 cells incubated with LPMix_LDL200 and additional treatment with sc-3088 or PMA (Figure 6J–M). Co-incubation with LPMix_LDL200 and sc-3088 led to lower *PML* mRNA and protein expression levels compared to the levels after incubation with the LPMix_LDL200 alone (mRNA: −55%, *p* < 0.05; Figure 6J; protein: −69%, *p* < 0.01; Figure 6L). In contrast, PMA co-treatment resulted in only non-significantly (*p* > 0.05) altered *PML* mRNA (Figure 6K) and PML protein levels (Figure 6M) compared to the levels measured after incubation with the LPMix_LDL200 alone.

Taken together, these results suggest that LDL-induced upregulation of PML is at least partially dependent on PKC activity.

### 2.7. The LDL-Induced Upregulation of IL-6 and IL-8 Expression in Endothelial Cells Is Regulated by a PKC-Mediated Increased Expression of PML

**Figure 7:**
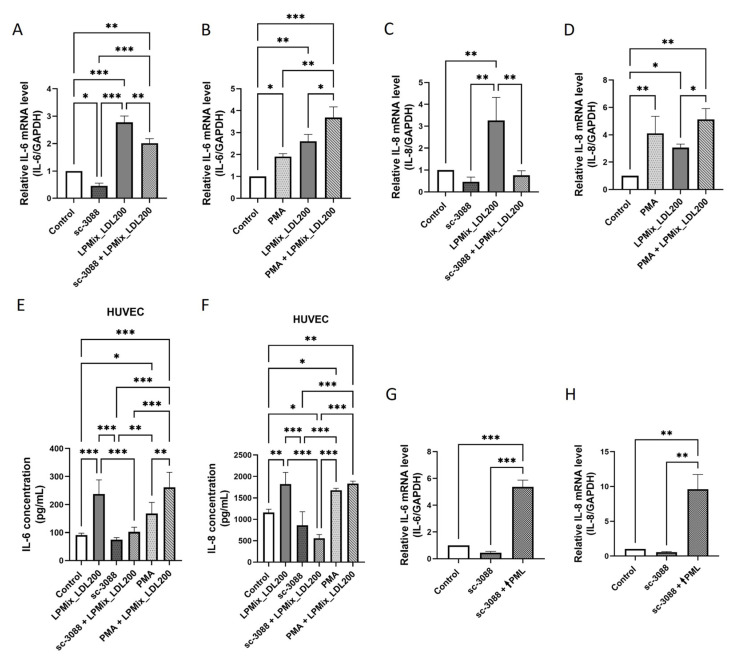
PKC influences LDL-triggered and PML-dependent IL-6 and IL-8 upregulation in endothelial cells. (**A**,**B**) RT-qPCR was performed on EA.hy926 cell extracts incubated with high LDL concentrations in LPMix with or without sc-3088 (1 µM) for 24 h to determine IL-6 mRNA (**A**) or IL-8 mRNA (**B**) levels. Expression values relative to control. *n* = 3, two-way ANOVA. (**C**,**D**) To determine IL-6 (**C**) or IL-8 mRNA (**D**) levels, RT-qPCR was performed on extracts of EA.hy926 cells incubated with high LDL concentrations in LPMix with or without PMA (80 nM) for 24 h. Expression values relative to control. *n* = 3, two-way ANOVA. (**E**,**F**) Concentrations of IL-6 (**E**) or IL-8 (**F**) protein in supernatants of HUVECs incubated with high LDL concentrations in LPMix with or without sc-3088 (1 µM) or PMA (80 nM) treatment for 24 h, as quantified by ELISA. *n* = 3, two-way ANOVA. (**G**,**H**) RT-qPCR for the quantification of IL-6 (**G**) or IL-8 (**H**) mRNA levels in EA.hy926 cells transfected with an EGFP-C1-PML-IV-vector in the absence/presence of sc-3088 (1 µM) for 24 h. Expression values relative to control. *n* = 3, two-way ANOVA All graphs shown as mean ± SD, * *p* < 0.05, ** *p* < 0.01, *** *p* < 0.001.

We then tested whether the expression of IL-6 and IL-8 is associated with PKC activity in ECs induced by LDL-exposure. Figure 7A,B show IL-6 mRNA levels in EA.hy926 cells incubated for 24 h with either sc-3088 or PMA in the presence or absence of LPMix_LDL200. Incubation with sc-3088 alone led to lower IL-6 mRNA levels (−54%, *p* < 0.05; Figure 7A), whereas incubation with PMA alone resulted in higher IL-6 mRNA levels (93%, *p* < 0.05; Figure 7B) compared to the levels in control cells. In contrast, incubation with the LPMix_LDL200 alone resulted in higher IL-6 mRNA levels (+177%, *p* < 0.001; Figure 7A), while incubation with the combination of LPMix_LDL200 and sc-3088 resulted in lower IL-6 mRNA levels (−28%, *p* < 0.01; Figure 7A). Simultaneous incubation of EA.hy926 cells with LPMix_LDL200 and PMA resulted in 42% higher IL-6 mRNA levels compared to cells treated with LPMix_LDL200 alone (*p* < 0.05; Figure 7B). Concordant results were found for the mRNA levels of IL-8 in EA.hy926 cells (Figure 7C,D) and IL-6 and IL-8 secretion in HUVECs (Figure 7E,F).

To find out whether PKC-mediated regulation of IL-6 and IL-8 expression depend on PML expression, EA.hy926 cells were either treated with sc-3088 alone for 24 h or additionally transfected with the pEGFP-C1-PML-IV vector (Figure 7G,H). Incubation of PML-transfected cells with sc-3088 resulted in 1061% higher (*p* < 0.001; Figure 7G) IL-6 and 1614% higher (*p* < 0.01; Figure 7H) IL-8 expression levels, respectively, compared to the levels in cells with sc-3088 treatment alone.

Taken together, these results indicate that the LDL-induced upregulation of IL-6 and IL-8 expression in ECs is positively related to the PKC-mediated PML upregulation.

## 3. Discussion

It was the major aim of this study to characterize the molecular mechanism(s) by which LDL triggers immunomodulatory responses in ECs. In the first step, we found that EA.hy926 cells or HUVECs actually express higher levels of the two interleukins IL-6 and IL-8 when stimulated with LDL. Using the two EC culture systems, it was subsequently demonstrated that: (1) LDL controls PML expression and PML-NB assembly, (2) PML mediates LDL-triggered upregulation of IL-6 and IL-8 expression, and (3) LDL increases PKC activity, which results in PML upregulation and subsequently higher IL-6 and IL-8 expression. Based on the combination of these experimental data, we conclude that LDL influences the secretion of IL-6 and IL-8 from ECs via PKC-mediated regulation of PML expression.

Lipoproteins are not only relevant for the transport of lipids throughout the vascular system, but also induce the transduction of signals in cells that finally trigger intracellular processes and influence the expression levels of target proteins, particularly associated with adhesion molecule expression on vascular ECs [21], chemokine receptor expression [22], and monocyte migration [23]. Studies with HUVECs and human umbilical artery endothelial cells revealed that LDL causes the activation of the transcription factor activator protein-1 (AP1) [24] and regulates the expression of vascular adhesion molecules such as vascular cell adhesion molecule-1 (VCAM-1) and E-selectin [21]. Furthermore, a significant increase in IL-6 levels after LDL exposure was observed in microvascular ECs [11], macrophages [25], and mesangial cells [26]. As we were particularly interested in characterizing the immunomodulatory responses of ECs to LDL exposure, we focused our molecular analysis on the expression patterns of IL-6 and IL-8. Indeed, we found an increase in mRNA expression and an increased secretion of IL-6 and IL-8 in EA.hy926 cells and HUVECs in a dose-dependent manner after LDL exposure. These results are consistent with data from the previous studies and suggest that EA.hy926 cells, in addition to HUVECs, are suitable to characterize the process of LDL induction of IL-6 and IL-8 expression in ECs.

Since the experimental silencing of *PML* expression was accompanied by lower synthesis rates of IL-1 and IL-6 in mouse embryonic fibroblasts and mouse macrophages [13,27], it was previously suggested that PML is a regulator of expression and secretion of these cytokines. Therefore, we examined whether PML expression in the two EC cultures used in this study is regulated after LDL exposure. RT-qPCR, immunoblotting, and immunocytochemistry consistently showed higher levels of *PML* mRNA and PML protein and a higher number of PML-NBs after only 3 h of incubation with an LPMix enriched with high concentrations of LDL. However, since it cannot be ruled out that other lipoproteins present in the LPMix influenced PML expression, the incubation of the ECs was additionally carried out with purified LDL and HDL in different concentrations. LDL in high concentrations led to stronger *PML* expression at the transcriptional level. These data suggest that LDL but not HDL is able to induce the upregulation of PML expression in ECs. However, since it has not been tested, it cannot be ruled out that VLDL and IDL also positively affect PML expression.

We also investigated whether PML contributes to LDL-induced regulation of IL-6 and IL-8 expression in the two EC cultures. Both overexpression by transfection with the *PML* gene and knockdown of *PML* with specific siRNAs demonstrated that PML as a downstream link in the signaling cascade mediates, at least in part, the positive regulatory effect of LDL on IL-6 and IL-8 expression.

Because a PKC-dependent signaling pathway is crucial for the production of pro-inflammatory cytokines [28], including IL-6 upregulation in monocytes [29], we then tested whether PKC is also involved in the regulation of the processes that cause PML activation and subsequent IL-6 and IL-8 upregulation in ECs in response to LDL exposure. For this purpose, PKC activity in the cell cultures was altered by incubation with chemical modulators, i.e., the established PKC activator PMA or the PKC inhibitor sc-3088. The experiments showed that: (1) LDL but not HDL dose-dependently increased PKC activity and, on the other hand, (2) PKC activity was associated with the rate of *PML* transcription and the numbers of PML-NBs. It was furthermore found that (3) the production and secretion of IL-6 and IL-8 were PKC-dependent in both EA.hy926 cells and HUVECs. This effect was also achieved with high LDL concentrations in the LPMix together with the PKC activity modulators. The combination of these findings leads to the conclusion that PKC is downstream of LDL and upstream of PML and IL-6/IL-8 upregulation in the signaling cascade in response to LDL exposure of ECs.

Taken together, the experimental findings of our study suggest that LDL triggers an increase of IL-6 and IL-8 expression in ECs (at least partially) via a PKC- and PML-mediated signal transduction cascade. It is conceivable that this process results in a pro-inflammatory environment in the arterial wall that not only causes the activation of additional ECs as a prerequisite of endothelial dysfunction, but also influences the functional integrity of other cells, especially macrophages and smooth muscle cells. Consequently, the novel signaling cascade described here could be of particular relevance for the occurrence of atherosclerosis.

By identifying the reactions in this signaling cascade and elucidating their sequence of occurrence, it is possible to speculate on the molecular mechanism by which this signaling cascade is realized and regulated. It is likely that the binding of LDL to one or more specific LDL receptors in the plasma membrane of ECs triggers transduction of specific intracellular signaling pathways. Several LDL receptors in ECs have been identified, including members of the LDL receptor family (LRP1, LRP5, LRP6) and members of the scavenger receptor family (SR-BI), as well as ALK1, a TGF-β-type receptor restricted to ECs [10,30]. Although none of these LDL receptors belong to the group of classical heptahelical receptors that lead to the activation of PKC [31], an interaction between LRP1 and PKC has already been described. When the expression of LRP1 was knocked down in chondrocytes, the amount of phosphorylated PKCζ was reduced [32]. Interestingly, a functional relationship between LDL and PKC has also been demonstrated in ECs without identifying the LDL receptor involved [33].

The PKC family consists of fifteen isoforms that are classified into conventional, novel, and atypical forms, which differ significantly in their biochemical profiles [34]. The three conventional PKC forms (PKCα, PKCß, and PKCy) require high Ca^2+^-concentrations, DAG, and phospholipids for their activation [34]. Interestingly, LDL exposure to ECs increases the cytosolic Ca^2+^-concentration [21], suggesting that one of these three conventional PKC forms transmits the information of LDL exposure into ECs. Indeed, Ren et al. demonstrated that the PKC-ß isoform is activated by LDL stimulation of HUVECs [33].

To date, a direct link between PKC and PML has not yet been reported, but one study showed that PKC activation resulted in PML-mediated SUMOylation of YAP1 [35], thus indirectly suggesting that there is (at least) an indirect PKC–PML interaction. Interestingly, sc-3088 is a peptide inhibitor corresponding to amino acids 19-31 of the pseudosubstrate domain expressed in the primary structures of the α, βI, βII, and γ isoforms of PKC and is significantly similar to stretches in the pseudosubstrate domains present in all novel and atypical PKC isoforms [36]. Furthermore, PMA activates both conventional and novel PKC isoforms by mimicking the activating ligand DAG [37]. Therefore, it is currently not possible to clearly distinguish whether only one or multiple PKC isoforms are involved in the regulation of PML. Interestingly, both PKC-dependent pathways and Ca^2+^-dependent mechanisms have been shown to play important roles in inducing the synthesis of pro-inflammatory cytokines [29,38,39]. Therefore, it is reasonable to speculate that LDL stimulates the production of pro-inflammatory cytokines in ECs at least partially via a Ca^2+^-dependent mechanism involving the activation of PKC.

For several reasons, it seems possible that the transcription factor nuclear factor kappa B (NF-kB) and/or AP-1 are involved in the transmission of information with regard to the activation of the upstream factors LDL, PKC, and PML to upregulate the transcription of the cytokines IL-6 and IL-8: (1) the expression of AP-1 and NF-kB is triggered by LDL [24,40], (2) AP1 and NF-kB are regulated by PKC [41,42], (3) a functional link between PML and NF-kB is already established [43,44], and (4) NF-kB and AP-1 control the expression of IL-6 and IL-8 by binding to specific DNA motifs within the promoter [45,46,47]. Interestingly, LDL in HUVECs appears to act predominantly through the AP-1 system [24], whereas other cell stimulators tend to regulate the transcription of their target genes via NF-κB.

OxLDL, which is a risk factor for atherosclerosis [48], has also been shown to stimulate PKC [49], influence NF-kB and AP-1 activity [50], and stimulate cytokine expression, including IL-6 and IL-8 [11,51,52,53]. To rule out the possibility that the effects observed in our system were caused by the presence of OxLDL, ELISA was performed, which showed that the OxLDL concentration in all lipoprotein eluates was very low and that the ratio of LDL to OxLDL was not significantly different between the LPMix and the purified lipoproteins, making it unlikely that our results were due to differences in the availability of OxLDL.

Controversial data have previously been published on the effect of LDL on the secretion of IL-8, as higher IL-8 secretion after LDL exposure was observed in human aortic smooth muscle cells but not in ECs [54]. This contradiction could be partly due to the different concentrations of LDL used. An effect of LDL not only on IL-6, as previously shown [11,24,25], but also on IL-8, is supported by the fact that the expression of both cytokines in ECs is regulated by the NF-kB pathway [55,56]. In addition, IL-8 is induced by intracellular Ca^2+^ mobilization [38,39,57], and the fact that LDL increases cytosolic Ca^2+^ in ECs [21,22,58,59] also argues for an increase of IL-8 in ECs.

Some biological and methodological constraints may limit the significance of our findings: (1) because LDL can still be oxidized in our experimental setup, it remains to be clarified whether this modification is responsible for at least some of the effects observed with presumably native LDL, and whether LDL thus plays a similar pro-inflammatory role as shown for OxLDL; (2) whether the LDL/PKC/PML pathway is also relevant in vivo; (3) whether this pathway affects other (vascular) cells such as macrophages and smooth muscle cells; and (4) whether the LDL/PKC/PML pathway is also present/active in these cells, which play an important role in the development of atherosclerosis.

In summary, the findings of this study show that elevated plasma levels of LDL trigger an immune-stimulating signaling cascade through its reaction with ECs. This interaction might contribute to the initiation of early inflammatory processes that lead to endothelial dysfunction and, in consequence, to the progression of atherosclerosis [60].

## 4. Materials and Methods

### 4.1. Lipoprotein Preparation

Blood lipoproteins were purified in collaboration with Prof. Dr. Britta Eickholt from the Institute of Biochemistry at the Charité–Universitätsmedizin Berlin and Prof. Dr. Michael Walter from the Institute for Clinical Chemistry and Laboratory Medicine at the University of Rostock.

The lipoproteins were isolated from the plasma of healthy volunteers (not under medication or dietary supplements, and starving for at least 10 h) via differential ultra-centrifugation at 40,000 rpm for 48 h at 4 °C according to their densities (VLDL; d = 0.095–1.006 g/mL, IDL; d = 1.006–1.019 g/mL, LDL; d = 1.019–1.063 g/mL, HDL; d = 1.063–1.21 g/mL) using a fixed-angle titanium rotor, TI-45 by Beckman Coulter (Indianapolis, IN, USA), in accordance with the methods described by Chisari et al. [61] and Leonhardt et al. [62]. After centrifugation, the supernatant was pipetted off and sterile-filtered (0.2 µm). LDL was desalted by gel filtration with an Econo-Pac 10 DG column (Bio-Rad, Hemel Hempstead, Hertfordshire, UK) using Krebs solution as eluent. For the determination of the lipoprotein concentrations, the Pierce BCA protein assay kit by Thermo Fisher Scientific (Waltham, MA, USA) was used.

### 4.2. Cell Culture

EA.hy926 cells were originally generated by fusing HUVECs with a thioguanine-resistant clone of adenocarcinoma human alveolar basal epithelial cells (A549). EA.hy926 cells (Elabscience, Houston, TX, USA) were cultured in Dulbecco’s modified Eagle’s medium (Gibco by Thermo Fisher Scientific, Waltham, MA, USA) containing 10% fetal bovine serum (Growth Medium, Gibco by Thermo Fisher Scientific, Waltham, MA, USA). HUVECs were isolated as previously described and cultured in an endothelial cell growth medium (PromoCell, Heidelberg, Germany). All cells were maintained at 37 °C in a humidified atmosphere of 5% CO_2_.

The approval for the isolation of HUVECs was obtained from the ethics committee of the Charité–Universitätsmedizin Berlin, Germany (EA4/107/17). Umbilical cords were obtained from healthy mothers with written informed consent in accordance with the Declaration of Helsinki.

### 4.3. Cell Stimulation Experiments with Lipoproteins

Three different carrier mixtures of lipoproteins (LPMix) in Krebs solution were used in the experiments, which all contained VLDL = 30 mg/dL, IDL = 10 mg/dL, and HDL = 50 mg/dL, but different concentrations for LDL. In the first mixture (LPMix_LDL50), low LDL levels were used (50 mg/dL), resulting in an LDL/HDL ratio of 1, whereas the second mixture (LPMix_LDL200) represented a pathophysiological assortment ratio with increased LDL levels (LDL = 200 mg/dL; LDL/HDL ratio = 4). The third mixture (LPMix_LDL100) corresponded to the physiological concentrations of lipoproteins in the human organism (LDL = 100 mg/dL; LDL/HDL ratio = 2). Isolated LDL was used in two concentrations, 200 mg/dL and 50 mg/dL, and isolated HDL was used in 100 mg/dL and 25 mg/dL. EA.hy926 cells or HUVECs were treated with the corresponding lipoproteins for 3 h or 24 h before cell harvesting and analysis.

### 4.4. Modulation of Cellular PKC Activity by sc-3088 and PMA

The PKC peptide inhibitor sc-3088 with the sequence FARKGALRQKNV (final concentration: 1 μM; Santa Cruz Biotechnology, Dallas, TX, USA) and the PKC activator PMA (final concentration: 80 nM; LC Laboratories, Woburn, MA, USA), both dissolved in dimethyl sulfoxide (DMSO), were added to the cell culture medium. DMSO served as a negative control.

### 4.5. Transfection with PML Expression Vector

Transfection of EA.hy926 cells with expression plasmids was performed as previously described [63]. Briefly, for transient transfection of the full-length *PML* gene into EA.hy926 cells, the pEGFP-C1-PML-IV plasmid was used in combination with the TurboFect reagent (Thermo Fisher Scientific, Waltham, MA, USA) according to the manufacturer’s instructions. Transfection efficiency was tested by RT-qPCR and by immunoblot analysis. The pEGFP-C1-PML-IV vector was a kind gift from PD Dr. Peter Hemmerich (Leibniz Institute for Ageing Research—Fritz Lipman Institute, Jena, Germany). The term “control cells” refers to cells transfected with the corresponding vector lacking a specific gene insert.

### 4.6. Transfection with Small Interfering RNA (siRNA)

Transfection of HUVECs was performed using a mixture of four unrelated siRNA species (25 nM final concentration each) directed against the *PML* nucleotide sequence (Catalog-ID: L-006547-00-0005C, Dharmacon, Lafayette, CO, USA). A non-gene-specific “scrambled” siRNA was used as a negative control. HUVECs were transfected using the transfection reagent interferin (Polyplus Transfection, Illkirch-Graffenstaden, France) according to the manufacturer’s instructions. Knockdown of the target mRNA was monitored 24 h after transfection with siRNAs by RT-qPCR and/or immunoblotting.

### 4.7. RNA Isolation, Reverse Transcription, and Real-Time PCR

RNA was extracted from EA.hy926 and HUVECs using the GeneMATRIX Universal RNA purification kit from EURx (Gdansk, Poland) according to the manufacturer’s instructions. Semi-quantitative RT-qPCR analysis was carried out using the QuantStudio 5 system (Applied Biosystems, Foster City, CA, USA)**.** Concentrations of specific cDNAs were determined using the GoTaq qPCR Master Mix (Promega Corporation, Madison, WI, USA). Primer sequences, product sizes, and annealing temperatures are listed in Table 1. Primers were purchased from BioTeZ Berlin-Buch GmbH (Berlin, Germany). In each experiment, a melting curve analysis was performed to verify that a single transcript was produced. RT-qPCR-relative mRNA levels were calculated using the comparative CT (2^−ΔΔ*C*T^) method, with GAPDH as a reference. The control of each experiment was set to 1. Non-RT and non-template controls were run for all reactions.

### 4.8. Immunoblotting

Cells were homogenized in RIPA buffer (Santa Cruz, Dallas, TX, USA) containing standard protease inhibitors (Sigma-Aldrich, Louis, MO, USA) for 15 min at 4 °C. Cell lysates were collected after centrifugation to be subjected to protein concentration determination using BCA protein assay kit (Thermo Fisher Scientific, Dallas, TX, USA). Total protein extracts (20 μg) were resolved by SDS-PAGE and transferred to nitrocellulose membranes (GE Healthcare, Chicago, IL, USA) for immunoblotting, as previously described [64]. Primary antibodies were diluted as follows: anti-PML (1:1000, Novus Biologicals, Littleton, CO, USA) and anti-GAPDH (1:10,000, Proteintech, Rosemont, IL, USA). The anti-PML polyclonal antibody used detects multiple isoforms of PML. All PML bands shown were analyzed for densitometric analysis. The control of each experiment was set to 1.

### 4.9. ELISA Assays and Activity Assays

IL-6 and IL-8 protein concentrations were determined in cell culture supernatants using commercial human ELISA kits (Invitrogen by Thermo Fisher Scientific, Waltham, MA, USA) according to the manufacturer’s guidelines. PKC activity was quantified using an assay kit purchased from Abcam (Cambridge, UK) according to the manufacturer’s guidelines. Samples were analyzed in triplicates. To determine the concentrations of OxLDL in the LPMix and purified LDL fractions, an OxLDL ELISA kit from Elabscience Biotechnology (Houston, TX, USA) was used.

### 4.10. Immunofluorescence Analysis

Cellular localization of PML was assessed on fixed EA.hy926 cells and HUVECs by immunocytochemistry implemented by confocal laser scanning microscopy, as previously described [63].

### 4.11. Statistical Analysis

Data were analyzed with GraphPad Prism 9 software (Boston, MA, USA) and presented as mean ± SD (*n* ≥ 3). All datasets were tested by Shapiro–Wilk for their normality of distribution prior to statistical analysis and the Brown–Forsythe test was performed to test the equality of variance. Comparisons between 2 groups were performed by a Student’s *t*-test (2-tailed unpaired), between >2 groups by one-way or by two-way ANOVA with Tukey’s multiple comparisons test.

## 5. Conclusions

In conclusion, our experimental data reveal that LDL influences the secretion of IL-6 and IL-8 from ECs via the PKC-mediated regulation of PML expression. This molecular cascade represents a previously unknown signaling pathway that might play a role in the response of ECs to high lipoprotein levels, e.g., to influence immunomodulatory processes in the vascular wall.

## Figures and Tables

**Table 1:** Primer sequences, product sizes, and annealing temperatures used to amplify the corresponding cDNA templates.

Template	Forward Primer	Reverse Primer	Product Size	Annealing Temperature
GAPDH	5′-ATG ACC TTG CCC ACA GCC TT-3′	5′-AAC TGC TTA GCA CCC CTG GC-3′	200 bp	60 °C
IL-6	5′-TGC CAG CCT GCT GAC GAA G-3′	5′-AGC TGC GCA GAA TGA GAT GAG-3′	77 bp	58 °C
IL-8	5′-ATG ACT TCC AAG CTG GCC GTG GC-3′	5′-TCT CAG CCC TCT TCA AAA ACT TCT C-3′	292 bp	58 °C
PML	5′-CCG CAA GAC CAA CAA CAT CTT-3′	5′-CAG CGG CTT GGA ACA TCC T-3′	91 bp	58 °C

## Data Availability

All data generated or analyzed during this study are included in this published article.

## Supplementary Materials

The following supporting information can be downloaded at: https://www.mdpi.com/article/10.3390/ijms24087306/s1.
