# Supplementary material for: LDL Affects the Immunomodulatory Response of Endothelial Cells by Modulation of the Promyelocytic Leukemia Protein (PML) Expression via PKC
Source: Int J Mol Sci. 2023 Apr 15;24(8):7306. doi: 10.3390/ijms24087306 (PMC10138343; doi:10.3390/ijms24087306)
Supplement: Supplementary file 1 [file ijms-24-07306-s001.zip › ijms-2305895-supplementary.pdf]

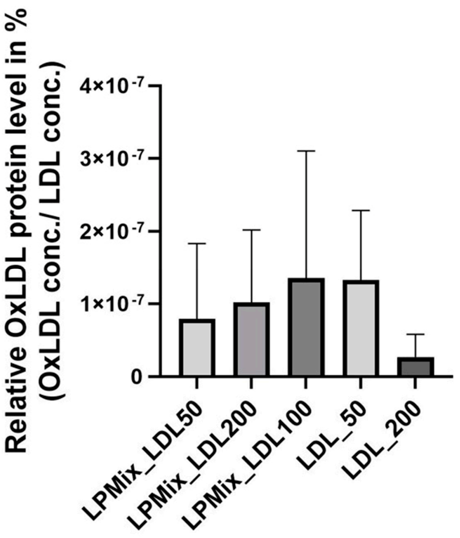

Figure S1. Relative OxLDL protein levels in LPMix and purified LDL fractions. ELISA was performed to determine OxLDL concentration in LPMix supplemented with LDL in different concentrations and with high or low concentrations of purified LDL. Expression values relative to LDL concentrations. $n = 4$. Graph is shown as mean ± SD.

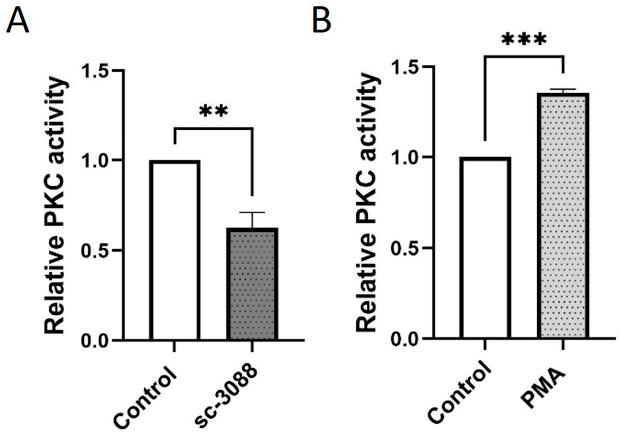

Figure S2. Effect of sc-3088 and PMA on PKC activity in endothelial cells. A, B ELISA was performed to determine PKC activity in EA.hy926 cell extracts either incubated with DMSO (control) or incubated with the PKC-peptide inhibitor sc-3088 (1 μM) (**A**) or the PKC activator PMA (80 nM) (**B**) for 24 h. $n = 3$, Student's *t*-test. All graphs shown as mean ± SD, ** $p < 0.01$, *** $p < 0.001$.
